# Mathematical modeling of glucose regulation and sleep resting heart rate to predict risk of metabolic dysregulation in cancer survivors

**DOI:** 10.1371/journal.pone.0339461

**Published:** 2026-01-02

**Authors:** Yue Liao, Christine Spadola, Souvik Roy

**Affiliations:** 1 Public Health Program, Department of Kinesiology, The University of Texas at Arlington, Arlington, Texas, United States of America; 2 School of Social Work, The University of Texas at Arlington, Arlington, Texas, United States of America; 3 Department of Mathematics, The University of Texas at Arlington, Arlington, Texas, United States of America; The First Hospital of Jilin University, CHINA

## Abstract

Sleep-related alterations in glucose metabolism and autonomic regulation play a critical role in the early development of type 2 diabetes mellitus (T2DM), especially among cancer survivors. Motivated by this interplay, we develop a physiologically informed mathematical model linking nocturnal glucose levels with resting heart rate dynamics. The model comprises of a coupled system of ordinary differential equations, and is fitted to wearable sensor data using particle swarm optimization. Sensitivity analysis is carried out to obtain the key parameters driving metabolic dysregulation, enabling the formulation of a personalized risk score. Successful validation using synthetic patient data and cancer survivor patient data suggests that this framework provides a non-invasive, interpretable tool for early T2DM risk stratification based on nocturnal physiological patterns.

## Introduction

There are more than 18 million cancer survivors in the US, and this number is expected to grow with advances in diagnostic and treatment capabilities [1]. Improved survival rates have highlighted the need to optimize the long-term health and well-being of cancer patients after treatment as part of their standard cancer care. Notably, the number of adult cancer survivors with comorbid diseases has increased substantially over the past two decades. Comorbidities can affect the life expectancy and survival of cancer patients, regardless of cancer stage and disease status. Therefore, optimal management of comorbid conditions and aggressive interventions for risk reduction can benefit the cancer survivor population.

Type 2 diabetes mellitus (T2DM) with hyperglycemia (high blood sugar) is a chronic metabolic disorder characterized by persistent high blood glucose levels, often due to insulin resistance and/or inadequate insulin production. T2DM is one of the most common comorbidities in cancer survivors and has been linked with increased cancer mortality [2,3]. Prediabetes is a critical stage in the progression towards T2DM, as individuals with prediabetes can effectively prevent or delay the onset of T2DM through lifestyle modifications [4]. Thus, it is important to identify at-risk individuals early to implement preventive measures to mitigate the growing burden of T2DM. Early detection is especially crucial for cancer patients because the prevalence of prediabetes rises significantly after cancer diagnosis and treatment, with rates increasing from 6% at diagnosis to over 21% later in the cancer continuum [5]. Since prediabetes is independently associated with an increased risk of several subsequent comorbidities, such as stroke, myocardial infarction, and chronic kidney disease [6–8], early detection and lifestyle intervention provide a window of opportunity to mitigate these potential long-term complications for cancer patients.

Although early detection of prediabetes is valuable, several challenges make it difficult. Prediabetes is often asymptomatic and, without screening, early insulin resistance can go unnoticed—about 80% of those affected are unaware of their condition [9]. Awareness is also low, with up to 93.7% unfamiliar with terms like “prediabetes” or “impaired fasting glucose,” and many mistakenly expecting diabetes-like symptoms [10]. Diagnostic inconsistencies further complicate detection; for instance, the American Diabetes Association defines prediabetes as fasting glucose ≥ 5.6 mmol/L [11], while the global threshold is > 6.1 mmol/L [12]. Standard one-time fasting glucose or HbA1c tests may also miss key indicators such as postprandial spikes, glycemic variability, or the dawn phenomenon—all of which independently raise the risk of developing T2DM [13–16]. Therefore, novel assessment methods and strategies are needed to facilitate the timely detection of signs of prediabetes and risks of progression toward T2DM and other potential complications for cancer patients.

With technological advancement in wearable devices, it is now much easier and more feasible to track an individual’s daily behaviors and biological metrics continuously over time. Fitness wristbands, smartwatches, and smart rings are now popular items in the consumer market, with a constant push to integrate more biological sensors to provide users with real-time information about their physiological and biochemical state, such as heart rate, glucose levels, and blood pressure. As we are able to collect more time-series data to learn about individuals’ changing behaviors and biological states, there is opportunity to leverage this information to improve our risk detection and prediction for chronic conditions such as T2DM.

Studies have shown a link between heart rate and glucose impairment. In [17,18], the authors did a cohort analysis to show the link between high resting heart rate and T2DM due to the impairment of the sympathetic nervous system. In [19], the study indicated that a lower heart rate variability was associated with a higher risk of diabetes. The study in [20] demonstrated that higher resting heart rate leads to a high risk of progression to diabetes. In [21], the authors show that impaired glucose leads to an elevated resting heart rate. The authors in [22] showed a strong and positive correlation between lower heart rate variability and glucose impairment during sleep. Moreover sleep behaviors, such as sleep variability can also impact glucose [23]. These studies indicate the interdependence of glucose metabolism with resting heart rate during sleep for accurate prediction of risk for metabolic dysregulation.

Mathematical modeling is a powerful asset in tackling the complex heterogeneity associated with metabolic dysregulation. The process of mathematical model development builds a theoretical framework for understanding the intricate relationship between various biological processes and the observable treatment outcomes in diabetes patients. This approach offers several benefits for improving recurrence prediction, future treatment optimization, and understanding disease progression in diabetes. In the context of predicting the risk of diabetes, mathematical modeling will provide several advantages over the traditional clinical studies that have been used for recurrence risk prediction. While the clinical studies are time-consuming, require lots of resources, and incur high costs, mathematical models can be developed relatively quickly and simulate a wide range of conditions, providing valuable insights without the need for large-scale trials [24,25]. Mathematical modeling will help incorporate crucial factors, such as effects of sleep patterns and heart rates on glucose metabolism, that cannot be directly observed and measured but can be inferred through the model’s predictions, allowing for a more comprehensive understanding of diabetes progression and informing individualized patient care.

There exist some computational models related to modeling glucose dynamics, diabetic risk, and heart rate dynamics. In [26], the authors use an ordinary differential equation (ODE) based model to analyze diabetic complications. The authors in [27] use a 28 variable ODE system to describe the pathophysiology of T2DM metabolism. An ODE model was developed to describe the glucose metabolism in patients and assess the efficacy of an anti-diabetic weight-reducing drug in [28]. The ODE model was comprised of 6 model variables involving 49 parameters. The authors in [29] developed an ODE model to study the progression of T2DM in patients with underlying obesity. In [30], the authors propose an ODE-based model to understand the effect of circadian rhythm on insulin secretion. A class of regression models was used to study the dynamics of heart rate deflection points by the authors in [31]. In [32], the authors develop a mathematical model of heart rate response to fluid perturbation. However, to date, no work exists that combines the resting heart rate dynamics with resting glucose dynamics during sleep to predict the risk of progression to diabetes.

In this paper, we develop a new mathematical model that integrates key biological processes that govern glucose metabolism and heart rate dynamics during sleep to predict the risk of diabetes development. We focus on glucose and heart rate data during sleep because of the link between sleep and glucose dysregulation, and the ease of tracking sleep using various options of wearable devices over a long period of time. Furthermore, our mathematical model is simple in the sense that it only tracks the dynamics of two model variables: glucose and heart rate, and contains significantly less number of parameters that make the model easy to implement and interpret. Finally, we validate our proposed model using glucose and heart rate data from cancer survivor patients and use a sensitivity analysis approach to compute a risk score for metabolic dysregulation. However, even though our model is validated on synthetic patients and cancer survivor patients, our model can also be equally applied to other patients for monitoring diabetes risk and progression, making our model versatile and robust.

The content of the paper is organized as follows: In the next section on materials and methods, we describe our proposed mathematical model, the theoretical analyses of our proposed model, the numerical methods used to implement the mathematical model and fit it to the measured datasets. The section on results presents the output of our model fits and formulation and computation of a risk score based on sensitivity analyses of the model parameters. We also present a discussion of future directions about potential improvements and extensions of our proposed model and end with a section of conclusions.

## Materials and methods

### Mathematical model

We consider a mathematical model that links sleep activity with glucose regulation. We first consider a model for the glucose-insulin dynamics: The plasma glucose dynamics can be modeled using the following ordinary differential equations (ODE)


$$\frac{dG}{dt}=R_{g}+\alpha_{G}G\frac{H-H_{sleep}}{H_{sleep}}-k_{g}\frac{G}{G+K_{g}}-E_{g}G+G_{A}\exp\left(-\frac{(t-t_{c}{)}^{2}}{2G_{w}^{2}}\right)\chi_{t\geq t_{c}}$$

The heart rate dynamics can be modeled using the following ODE


$$\frac{dH}{dt}=-\beta_{H}(H-H_{\text{sleep}})+H_{v}\frac{G-G_{\text{sleep}}}{G_{\text{sleep}}}$$

The glucose–heart rate interaction model represents a physiologically grounded system of coupled ordinary differential equations that describe how glucose dynamics influence heart rate regulation over time. The glucose equation includes several key terms reflecting distinct biological processes. First, the basal production rate *R*_*g*_ accounts for endogenous glucose generation, such as hepatic gluconeogenesis and glycogenolysis. Glucose is removed from the bloodstream via two mechanisms: a nonlinear uptake term $\frac{G}{G+K_{g}}k_{g}$, which models tissue-level glucose absorption following Michaelis–Menten kinetics, and a linear clearance term *E*_*g*_*G*, representing metabolic decay and renal excretion. The Michaelis–Menten component captures transporter saturation behavior, when glucose is low, uptake increases nearly linearly; when high, uptake plateaus due to limited transporter capacity. It also reflects the saturable, insulin-mediated uptake of glucose by peripheral tissues, consistent with Michaelis–Menten kinetics commonly used to describe insulin-dependent transport [33]. To capture transient physiological effects upon waking, the model includes a Gaussian-shaped input term $G_{A}\exp\left(-\frac{(t-t_{c}){\;}^{2}}{2G_{w}^{2}}\right)$ (see [28,30] for similar spike terms due to food intake). This term serves as an exogenous stimulus that temporarily elevates glucose levels at a specific circadian phase.

The heart rate equation is similarly composed of intrinsic and glucose-driven components. The term $-\beta_{H}(H\;-\;H_{\text{sleep}})$ governs natural relaxation toward a resting heart rate $H_{\text{sleep}}$, with decay rate $\beta_{H}$ modeling parasympathetic influence. The interaction term $H_{v}\frac{G-G_{\text{sleep}}}{G_{\text{sleep}}}$ reflects the excitatory effect of glucose on heart rate, mediated by sympathetic nervous system activation. When glucose rises above its baseline $G_{\text{sleep}}$, this term becomes positive, accelerating the heart rate; when below baseline, it acts to suppress heart rate. The sensitivity of this interaction is controlled by the coupling strength $H_{v}$, and the normalization by $G_{\text{sleep}}$ ensures interpretability across individuals. The combined ODE model is given below along with the description of the parameters in Table 1.

$$\frac{dG}{dt}=R_{g}+\alpha_{G}G\frac{H-H_{sleep}}{H_{sleep}}-k_{g}\frac{G}{G+K_{g}}-E_{g}G+G_{A}\exp\left(-\frac{(t-t_{c}{)}^{2}}{2G_{w}^{2}}\right)\chi_{t\geq t_{c}},\;\frac{dH}{dt}=-\beta_{H}(H-H_{\text{sleep}})+H_{v}\frac{G-G_{\text{sleep}}}{G_{\text{sleep}}}.$$ (1)

**Table 1:** Glucose-heart rate model variables and parameters.

Variables	Description	Units
*G*(*t*)	Plasma glucose concentration	mg/dl
*H*(*t*)	Heart rate	Beats per minute (BPM)
*t*	Time	min
*R*_*g*_	Basal glucose production rate	mg/dl/min
$\alpha_{G}$	Modulation coefficient for glucose by heart rate	min^−1^
*K*_*g*_	Glucose transporter saturation level	mg/dl
*k*_*g*_	Rate of glucose consumption gain	min^−1^
*E*_*g*_	Rate of glucose clearance	min^−1^
$\beta_{H}$	HR decay rate towards resting HR	min^−1^
$H_{v}$	Coupling strength of glucose to heart rate	BPM/min
$G_{\text{sleep}}$	Baseline glucose concentration during sleep	mg/dl
$H_{\text{sleep}}$	Baseline HR during sleep	BPM
*G*_*A*_	Magnitude of transient glucose spike	mg/dl/min
*G*_*w*_	Duration of the transient glucose spike	min
*t*_*c*_	Center time of transient spike	min

### Theoretical analysis

#### Positivity, existence, and uniqueness.

For the following analyses, we write the ODE system (1) in a compact form as follows:

$$\frac{dX}{dt}=F(X,\theta )+G(t,\theta ),\;t\in (0,T),\;X(0)=X_{0},$$ (2)

where


$$X=[G,H],\;F=[R_{g}+\alpha_{G}x_{1}(x_{2}/H_{sleep}-1)-k_{g}x_{1}/(x_{1}+K_{g})-E_{g}x_{1},\;-\beta_{H}(x_{2}-H_{sleep})+H_{v}(x_{1}/G_{sleep}-1)],\;\theta =[R_{g},k_{g},K_{g},E_{g},G_{A},G_{w},\beta_{H},H_{v}],\;G=[G_{A}\exp(-(t-t_{c}{)}^{2}/(2G_{w}^{2}))\chi_{t\geq t_{c}},0].$$

**Lemma 1.** *The solutions of (2) are non-negative in the sense that if $X_{0}\geq 0$, we have X(t)≥0 for all t∈[0,T].*

*Proof:* We can write (2) in a productive-destructive form as follows:

$$\frac{dX}{dt}=P(X,\theta )-D(X,\theta )X+G(t,\theta ),$$ (3)

where P,D,G are positive, i.e., if X,θ≥0, we have P,D,G≥0, componentwise. Consider the integrating factor vector I=exp(∫D dt). The (3) can be rewritten as

$$\frac{d(IX)}{dt}=I[P(X,\theta )+G(t,\theta )].$$ (4)

Since, $X_{0}\geq 0,$ we have $IX_{0}\geq 0$. Thus, (4) gives us that IX(t)≥0 for all t∈[0,T]. Since, I>0, we have that X(t)≥0 for all t∈[0,T]. ◻

We next prove the boundedness of the solution of (2).

**Lemma 2.** *A solution X=[G,H] of (2) is bounded.*

*Proof:* To show that the solutions *G*(*t*) and *H*(*t*) remain bounded for all t∈[0,T], we define a Lyapunov-like function


V(G,H)=G+aH

for some constant *a* > 0 to be chosen. Computing the derivative of *V* gives us


$$\frac{dV}{dt}=\frac{dG}{dt}+a\frac{dH}{dt}$$

$$=R_{g}+\alpha_{G}G\frac{H-H_{\text{sleep}}}{H_{\text{sleep}}}-\frac{G}{G+K_{g}}k_{g}-E_{g}G+a\left[-\beta_{H}(H-H_{\text{sleep}})+H_{v}\frac{G-G_{\text{sleep}}}{G_{\text{sleep}}}\right]$$

We choose $a=\frac{\alpha_{G}G_{\text{sleep}}}{H_{v}H_{\text{sleep}}}$ so that the positive terms involving *GH* and *G* are grouped. Then, for large values of *G* and *H*, we obtain


$$\frac{dV}{dt}\leq -\left(\frac{k_{g}}{2}+E_{g}\right)G-\frac{a\beta_{H}}{2}H+C$$

where *C* is a constant depending on system parameters. Thus, $\frac{dV}{dt}<0$ outside a compact level set of *V*. Therefore, the solutions *G*(*t*) and *H*(*t*) remain bounded for all t∈[0,T]. ◻

We now state and prove the existence and uniqueness of solutions of (2).

**Theorem 1.** *There exists an unique solution X of (2) in (H*^*1*^*(0,T))*^*2*^.

*Proof:* From Lemma 2, we have that X is bounded. This implies that F satisfies the following conditions:

1. F is continuous with respect to X.
2. F is measurable with respect to *t*.
3. F is bounded.
4. The derivative of F with respect to X is also bounded.

Thus, F satisfies the Caratheodory’s conditions, which gives the existence and uniqueness of a solution $X\in (H^{1}(0,T)){\;}^{2}$ of (2). ◻

#### Equilibrium points and stability analysis.

To discuss the asymptotic behavior of our model, we consider the absence of the spike term, i.e., *G*_*A*_ = 0. At equilibrium, setting $\frac{dH}{dt}=0$ gives us


$$-\beta_{H}(H-H_{\text{sleep}})+H_{v}\frac{G-G_{\text{sleep}}}{G_{\text{sleep}}}=0.$$

Rearranging,


$$\beta_{H}(H-H_{\text{sleep}})=H_{v}\frac{G-G_{\text{sleep}}}{G_{\text{sleep}}}.$$

Thus,

$$H=H_{\text{sleep}}+\frac{H_{v}}{\beta_{H}}\frac{G-G_{\text{sleep}}}{G_{\text{sleep}}}.$$ (5)

Setting $\frac{dG}{dt}=0$ without the transient term, we obtain


$$R_{g}+\alpha_{G}G\frac{H-H_{\text{sleep}}}{H_{\text{sleep}}}-\frac{G}{G+K_{g}}k_{g}-E_{g}G=0.$$

Substituting for *H*, we get


$$R_{g}+\alpha_{G}G\left(\frac{1}{H_{\text{sleep}}}\times \frac{H_{v}}{\beta_{H}}\times \frac{G-G_{\text{sleep}}}{G_{\text{sleep}}}\right)-\frac{G}{G+K_{g}}k_{g}-E_{g}G=0.$$

Simplifying,


$$R_{g}+\frac{\alpha_{G}H_{v}}{\beta_{H}H_{\text{sleep}}G_{\text{sleep}}}G(G-G_{\text{sleep}})-\frac{G}{G+K_{g}}k_{g}-E_{g}G=0.$$

Define

$$A=\frac{\alpha_{G}H_{v}}{\beta_{H}H_{\text{sleep}}G_{\text{sleep}}}.$$ (6)

This implies


$$R_{g}+AG(G-G_{\text{sleep}})-\frac{G}{G+K_{g}}k_{g}-E_{g}G=0.$$

The above equation can be written as


$$AG^{3}+BG^{2}+CG+D=0,$$

where


$$A=\frac{\alpha_{G}H_{v}}{\beta_{H}H_{\text{sleep}}G_{\text{sleep}}},\;B=AK_{g}-AG_{\text{sleep}}-E_{g},\;C=R_{g}+AK_{g}G_{\text{sleep}}-E_{g}K_{g}-k_{g},\;D=R_{g}K_{g}.$$

This gives us


$$G=\sqrt[{3}]{-\frac{1}{2}\left(\frac{D}{A}+\frac{2B^{3}}{27A^{3}}-\frac{BC}{3A^{2}}\right)+\sqrt{{\left(\frac{1}{2}\left(\frac{D}{A}+\frac{2B^{3}}{27A^{3}}-\frac{BC}{3A^{2}}\right)\right)}^{2}+{\left(\frac{1}{3}\left(\frac{C}{A}-\frac{B^{2}}{3A^{2}}\right)\right)}^{3}}}\;+\sqrt[{3}]{-\frac{1}{2}\left(\frac{D}{A}+\frac{2B^{3}}{27A^{3}}-\frac{BC}{3A^{2}}\right)-\sqrt{{\left(\frac{1}{2}\left(\frac{D}{A}+\frac{2B^{3}}{27A^{3}}-\frac{BC}{3A^{2}}\right)\right)}^{2}+{\left(\frac{1}{3}\left(\frac{C}{A}-\frac{B^{2}}{3A^{2}}\right)\right)}^{3}}}-\frac{B}{3A},$$

Since, we do not have closed-form solutions for *G* from the aforementioned equations, we will use the Descartes rule of signs to deduce some information about the roots. Only under the condition that *B*,*C* have opposite signs, there can be either two or none positive roots. This implies


$$AG_{sleep}+E_{g}<AK_{g}<E_{g}K_{g}-k_{g}-R_{g},\;\text{ or }E_{g}K_{g}-k_{g}-R_{g}<AK_{g}<AG_{sleep}+E_{g}.$$

For the stability analysis, we define the Jacobian matrix *J* of the system as follows:

$$J=[\begin{array}{cc} \alpha_{G}\frac{H^{*}-H_{\text{sleep}}}{H_{\text{sleep}}}-\frac{k_{g}K_{g}}{(G^{*}+K_{g}{)}^{2}}-E_{g} & \alpha_{G}\frac{G^{*}}{H_{\text{sleep}}}\\ \frac{H_{v}}{G_{\text{sleep}}} & -\beta_{H} \end{array}],$$ (7)

where (G^*^,H^*^) denote the equilibrium points obtained from the previous section. Then the equilibrium $\left(G^{*},H^{*}\right)$ is locally asymptotically stable if


$$\text{Trace}(J)=\alpha_{G}\frac{H^{*}-H_{\text{sleep}}}{H_{\text{sleep}}}-\frac{k_{g}K_{g}}{(G^{*}+K_{g}{)}^{2}}-E_{g}-\beta_{H}<0$$

$$\text{Det}(J)=\left(\alpha_{G}\frac{H^{*}-H_{\text{sleep}}}{H_{\text{sleep}}}-\frac{k_{g}K_{g}}{(G^{*}+K_{g}{)}^{2}}-E_{g}\right)(-\beta_{H})-\left(\alpha_{G}\frac{G^{*}}{H_{\text{sleep}}}\cdot \frac{H_{v}}{G_{\text{sleep}}}\right)>0$$

Using the value of H^*^ obtained above as


$$H^{*}=H_{\text{sleep}}+\frac{H_{v}}{\beta_{H}}\cdot \frac{G^{*}-G_{\text{sleep}}}{G_{\text{sleep}}},$$

we have the equilibrium $\left(G^{*},H^{*}\right)$ is locally asymptotically stable if


$$\text{Trace}(J)=\alpha_{G}\cdot \frac{H_{v}(G^{*}-G_{\text{sleep}})}{\beta_{H}H_{\text{sleep}}G_{\text{sleep}}}-\frac{k_{g}K_{g}}{(G^{*}+K_{g}{)}^{2}}-E_{g}-\beta_{H}<0$$

$$\text{Det}(J)=\left(\alpha_{G}\cdot \frac{H_{v}(G^{*}-G_{\text{sleep}})}{\beta_{H}H_{\text{sleep}}G_{\text{sleep}}}-\frac{k_{g}K_{g}}{(G^{*}+K_{g}{)}^{2}}-E_{g}\right)(-\beta_{H})-\alpha_{G}\cdot \frac{G^{*}H_{v}}{H_{\text{sleep}}G_{\text{sleep}}}>0$$

We define the following term


$$\gamma =\frac{\alpha_{G}H_{v}}{\beta_{H}H_{\text{sleep}}G_{\text{sleep}}}.$$

The condition Trace(J)<0 will be satisfied if


$$\gamma (G^{*}-G_{\text{sleep}})-E_{g}-\beta_{H}<0,$$

since


$$\frac{k_{g}K_{g}}{(G^{*}+K_{g}{)}^{2}}\geq 0.$$

This gives us the following condition


$$G^{*}<G_{\text{sleep}}+\frac{E_{g}+\beta_{H}}{\gamma }$$

The condition Det(J)<0 will be satisfied if


$$-\beta_{H}\left(\gamma (G^{*}-G_{\text{sleep}})-E_{g}\right)>\alpha_{G}\cdot \frac{G^{*}H_{v}}{H_{\text{sleep}}G_{\text{sleep}}}$$

This gives us


$$G^{*}<\frac{G_{\text{sleep}}}{2}+\frac{E_{g}}{2\gamma }$$

Thus, for asymptotic stability, we require


$$G^{*}<\min\left\{G_{\text{sleep}}+\frac{E_{g}+\beta_{H}}{\gamma },\frac{G_{\text{sleep}}}{2}+\frac{E_{g}}{2\gamma }\right\}$$

**Remark 1.** *The system we analyzed above assumes that the spike term is absent, i.e., G*_*A*_* = 0, to study the autonomous equilibrium dynamics. However, in the original model, this term represents a possible dawn phenomenon glucose spike. It renders the system non-autonomous, and hence a true equilibrium point, in the strict mathematical sense, does not exist when $G_{A}\neq 0$. Thus, to analyze long-term behavior and stability, we study the behavior of the autonomous system, which corresponds to the state of the system either before or long after the transient stimulus has occurred. Under this approximation, the equilibrium point (G*^*^,H^*^) represents the physiological baseline during sleep, with the spike acting as a perturbation that momentarily shifts the system away from equilibrium. The stability analysis guarantees that, provided the eigenvalues of the Jacobian are negative, the system will return to the equilibrium point after the spike fades. Thus, the spike term does not alter the equilibrium itself but induces a transient excursion around it. If the equilibrium point is locally asymptotic stable, it ensures that such excursions decay over time, restoring homeostasis.

### Numerical methods

#### Synthetic data.

We first consider synthetic data cases and demonstrate that our model also provides reasonable inference for such cases. To generate the synthetic data, we simulate the ODE system (1) in the time interval [0,600], in 5 minute intervals, with certain values of the parameters and the initial conditions and then add Poisson noise to the data using values drawn from a Poisson distribution with the distribution parameter λ=1. This mimics the realistic scenario where the data does have random variations across time.

#### Real data preprocessing.

We next consider real-life data collected by commercially available wearable devices from four female breast cancer patients with different clinical risk predictions. All patients wore a continuous glucose monitor (Freestyle Libre) and a Fitbit wristband with a heart rate sensor continuously for 4 weeks. All patients have completed primary treatment at the time of data collection. To prepare the glucose and heart rate time series for model fitting, we performed a multi-stage data processing and smoothing pipeline. The objective was to extract high-resolution, temporally aligned, and noise-reduced signals representing nocturnal glucose and heart rate dynamics for a representative subject.

The first case was from My Moves, a physical activity intervention study for cancer survivors conducted between March 2020 to August 2021. My Moves was approved by The University of Texas MD Anderson Cancer Center Institutional Review Board (protocol #: 2018-0299). The recruitment period was between 10/03/2020 - 03/02/2021. The rest of the three cases were from Project KNOWN, an ongoing physical activity intervention study for cancer survivors started in April 2022 (https://journals.plos.org/plosone/article?id=10.1371/journal.pone.0274492). Project KNOWN was approved by the University of Texas at Arlington’s Institutional Review Board (protocol #: 2022–0177). The recruitment period was between 01/03/2023 - 30/07/2025. All study participants provided written informed consent.

The raw glucose dataset consisted of timestamped measurements recorded at irregular (usually 5 min) intervals. From this dataset, only nighttime data spanning from 9:00 PM to 9:00 AM were extracted. Each timestamp was converted to a consistent datetime format, and invalid or missing values were removed. For each night in the dataset, we constructed a uniformly spaced temporal grid ranging from 11:30 PM to 8:36 AM the following day, with a fixed interval of five minutes. A linear interpolation procedure was then applied to map the irregularly sampled glucose measurements onto this uniform grid. This procedure was repeated for each night, and the interpolated daily time series were concatenated.

After interpolating individual nights, we computed a pointwise average across all days to produce a representative nocturnal glucose trajectory. Averaging was performed at each time point in the common grid, yielding a smooth composite profile. To ensure temporal consistency, the time axis was reparameterized by computing the number of minutes since a fixed baseline time of 11:30 PM. This reparameterization aligned all measurements on a continuous time axis from zero to the endpoint, thus facilitating downstream numerical simulation and model fitting.

A similar procedure was followed for heart rate data. The original heart rate recordings were sampled at one-minute intervals. As with glucose, data points outside the 9:00 PM to 9:00 AM window were discarded. For each night, the remaining values were interpolated onto the same five-minute grid using linear interpolation. The nightly interpolated heart rate series were aggregated, and the pointwise average was computed to obtain a mean nocturnal heart rate curve. The time axis was then translated using the same reference point as in the glucose processing, resulting in a temporally aligned dataset.

Following temporal alignment and averaging, both glucose and heart rate signals were subjected to a final smoothing step using the Savitzky-Golay filter. This technique fits successive subsets of the data with low-degree polynomials via least-squares regression, allowing for smooth approximation while preserving local trends and extrema. We used a window length of 31 points and a third-order polynomial for both signals. The smoothed trajectories were plotted alongside the raw averages to visually confirm the suppression of measurement noise and high-frequency fluctuations.

#### Parameter estimation.

To estimate the parameters governing the glucose-heart rate dynamic model, we formulated a constrained optimization problem centered on minimizing a data misfit loss function. The goal was to infer a biologically meaningful parameter vector that enables the system of ODEs to reproduce the observed glucose and heart rate time series. The loss function ℒ(θ) quantifies the discrepancy between the model-predicted and observed measurements of glucose and heart rate. It is defined as the sum of mean squared errors (MSE) over the full time horizon

$$ℒ(\theta )=\frac{1}{N}\sum_{i=1}^{N}{\left(G(t_{i};\theta )-G_{\text{obs}}(t_{i})\right)}^{2}+\frac{1}{N}\sum_{i=1}^{N}{\left(H(t_{i};\theta )-H_{\text{obs}}(t_{i})\right)}^{2},$$ (8)

where

- $\theta \in {\mathbb{R}}^{9}$ is the parameter vector,
- $G(t_{i};\theta )$ and $H(t_{i};\theta )$ are the glucose and heart rate values simulated by the model at time *t*_*i*_,
- $G_{\text{obs}}(t_{i})$ and $H_{\text{obs}}(t_{i})$ are the observed values at time *t*_*i*_,
- *N* is the number of data points.

Such optimization frameworks have been used successfully in context of maximum-likelihood parameter estimation problems [34,35] and tomographic inverse problems [36–39]. The ODE system is integrated using MATLAB’s ode45 solver with stringent tolerances: a relative tolerance of 10^−6^, an absolute tolerance of 10^−8^, and a constraint on maximum step size to accurately resolve transient features in the dynamics. Integration proceeds from the initial time to the final observation time using initial conditions derived from the first observed values of glucose and heart rate. To align model output with observational data, simulation results were interpolated onto the discrete time points of the measurements using linear interpolation. Each parameter is bounded within a feasible range to guide the optimizer as given in Table 2.

**Table 2:** Ranges of parameters. Corresponding lower and upper bounds of parameters in the proposed mathematical model.

Parameter	Lower Bound	Upper Bound
*R*_*g*_	0.001	10
$\alpha_{g}$	0.001	0.1
*k*_*g*_	0.001	10
*K*_*g*_	1	10
*E*_*g*_	0.001	5
$\beta_{H}$	0.001	1
$H_{v}$	0.001	30
*G*_*A*_	0.01	20
*G*_*w*_	10	300

The loss function is minimized using particle swarm optimization (PSO), which is a population-based, stochastic optimization algorithm that draws inspiration from the coordinated behavior observed in natural systems such as bird flocking and fish schooling. It is particularly well-suited for problems involving nonlinear, non-convex, or non-differentiable objective functions. In the present study, PSO was employed to minimize the loss function associated with fitting a dynamical model of glucose and heart rate to time-resolved observational data. The parameter vector to be estimated consists of nine physiological quantities, and the optimization is conducted over a bounded domain for each parameter.

In the PSO framework, a collection of candidate solutions, referred to as particles, is initialized randomly within the search space. Each particle represents a position vector in the parameter space and carries an associated velocity vector that determines its direction and magnitude of movement at each iteration. The position of a particle is updated iteratively based on three contributing terms: its current velocity, the best position it has encountered in its own history, and the best position found by the entire swarm. This mechanism enables the particles to explore the search space collaboratively, balancing exploration and exploitation.

At each iteration, the velocity of a particle is updated as a weighted combination of its previous velocity, the difference between its current position and its personal best position, and the difference between its current position and the global best position known to the swarm. Mathematically, this is expressed as


$$v_{i}^{\left(t+1\right)}=wv_{i}^{\left(t\right)}+c_{1}r_{1}\left(p_{i}^{\text{best}}-x_{i}^{\left(t\right)}\right)+c_{2}r_{2}\left(g^{\text{best}}-x_{i}^{\left(t\right)}\right),$$

$$x_{i}^{\left(t+1\right)}=x_{i}^{\left(t\right)}+v_{i}^{\left(t+1\right)},$$

where $x_{i}^{\left(t\right)}$ and $v_{i}^{\left(t\right)}$ denote the position and velocity of particle *i* at iteration *t*, respectively. The terms $p_{i}^{\text{best}}$ and $g^{\text{best}}$ represent the best positions found so far by particle *i* and by the entire swarm. The constants *c*_1_ and *c*_2_ are positive acceleration coefficients, and *r*_1_ and *r*_2_ are random variables uniformly distributed in the interval [0,1], which introduce stochasticity into the search dynamics. The parameter *w* is the inertia weight that controls the balance between global exploration and local exploitation.

For our problem, the PSO algorithm was initialized with a swarm of 60 particles. The algorithm was executed for a maximum of 300 iterations. The particles were constrained to remain within the prescribed parameter bounds throughout the optimization process. For each candidate parameter vector generated during the optimization, the associated loss function was evaluated by numerically integrating the system of ordinary differential equations with those parameters and comparing the resulting trajectories to the measured glucose and heart rate data. If a candidate parameter set resulted in a failure of the numerical solver, produced non-finite values, or yielded incomplete integration, the corresponding loss was set to a large penalty value to discourage further exploration in that region of the parameter space.

To maintain numerical stability and reproducibility, the optimization was performed in a serial computing environment. Although the implementation allowed for a subsequent local refinement step using gradient-based optimization, in practice, the global optimization results obtained via PSO were found to be sufficient for producing accurate fits with interpretable physiological parameters. The final parameter vector was chosen as the position of the globally best particle after convergence, and this solution was used for subsequent model simulation and analysis.

### Ethics statement

Data in this paper were from a study that was reviewed and approved by The University of Texas MD Anderson Cancer Center Institutional Review Board (protocol #:2018-0299), and another study that was reviewed and approved by The University of Texas at Arlington’s Institutional Review Board (protocol #: 2022–0177). All study participants provided written informed consent.

## Results

We now present some numerical simulations to demonstrate the applicability of our model to risk-stratify diabetic stages. To illustrate the potential application of our proposed model, we applied the model to two synthetic patient data test cases and to real-life data from four cancer survivors with different diabetes risk profiles, based on their fasting glucose and HbA1c values. The six patients collectively represent the continuum of T2DM risk, spanning from individuals at normal risk to those with a clinical diagnosis of T2DM. We also determine the quality of our model fits through the following relative discrete *l*^2^ error percentage


$$\|A{\|}_{r}=\frac{\|A_{mod}-A_{obs}{\|}_{l_{2}}}{\|A_{obs}{\|}_{l_{2}}}\times 100\%,$$

where *A*_*mod*_ is the output fit obtained from the ODE model (1) and *A*_*obs*_ is the observed data.

**Fig 1:**
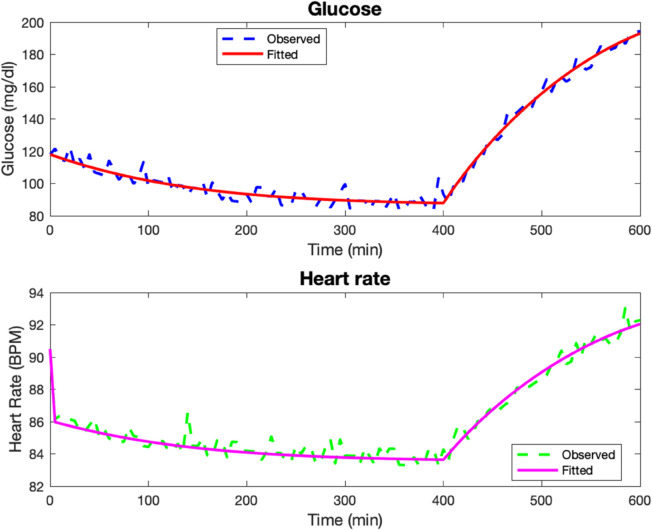
Test Case 1. Data and model fits for a synthetic patient 1.

In the first test case, the parameters used to generate the data are as follows: $R_{g}=8.2,\alpha_{g}=0.001,k_{g}=9,K_{g}=9,E_{g}=0.001,\beta_{H}=0.6,H_{v}=5,G_{A}=1,$ $G_{W}=250,G_{0}=118,H_{0}=90,G_{sleep}=105,H_{sleep}=90$, before Poisson noise is added to the data as described the Numerical methods section. The model fits are given in Fig 1. The computed parameters are $R_{g}=9.165,\alpha_{g}=0.002,k_{g}=10,K_{g}=8.904,E_{g}=0.001,\beta_{H}=0.579,H_{v}=3.893,G_{A}=0.974,G_{W}=263.86$.

The relative discrete *l*^2^ error percentages for the glucose and heart rate fits are $\|G{\|}_{r}=3.97\%,\;\|H{\|}_{r}=0.58\%$.

**Fig 2:**
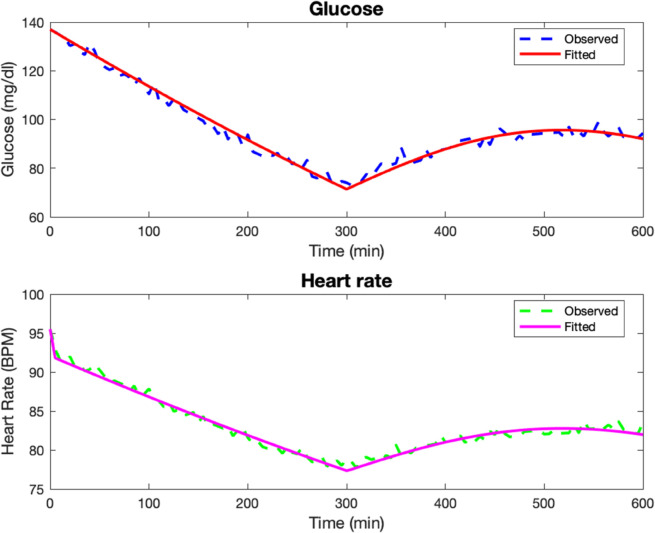
Test Case 2. Data and model fits for a synthetic patient 2.

In the second test case, the parameters used to generate the data are as follows: $R_{g}=7.5,\alpha_{g}=0.01,k_{g}=8,K_{g}=5,E_{g}=0.001,\beta_{H}=0.613,H_{v}=15,G_{A}=0.306,$ $G_{W}=300,G_{0}=135,H_{0}=95,G_{sleep}=105,H_{sleep}=85$, before Poisson noise is added to the data as described the Numerical methods section. The model fits are given in Fig 2. The computed parameters are $R_{g}=0.001,\alpha_{g}=0.001,k_{g}=0.139,K_{g}=10,E_{g}=0.001,\beta_{H}=0.001,H_{v}=1.753,G_{A}=0.381,G_{W}=203.04$.

The relative discrete *l*^2^ error percentages for the glucose and heart rate fits are $\|G{\|}_{r}=2.16\%,\;\|H{\|}_{r}=0.68\%$.

**Fig 3:**
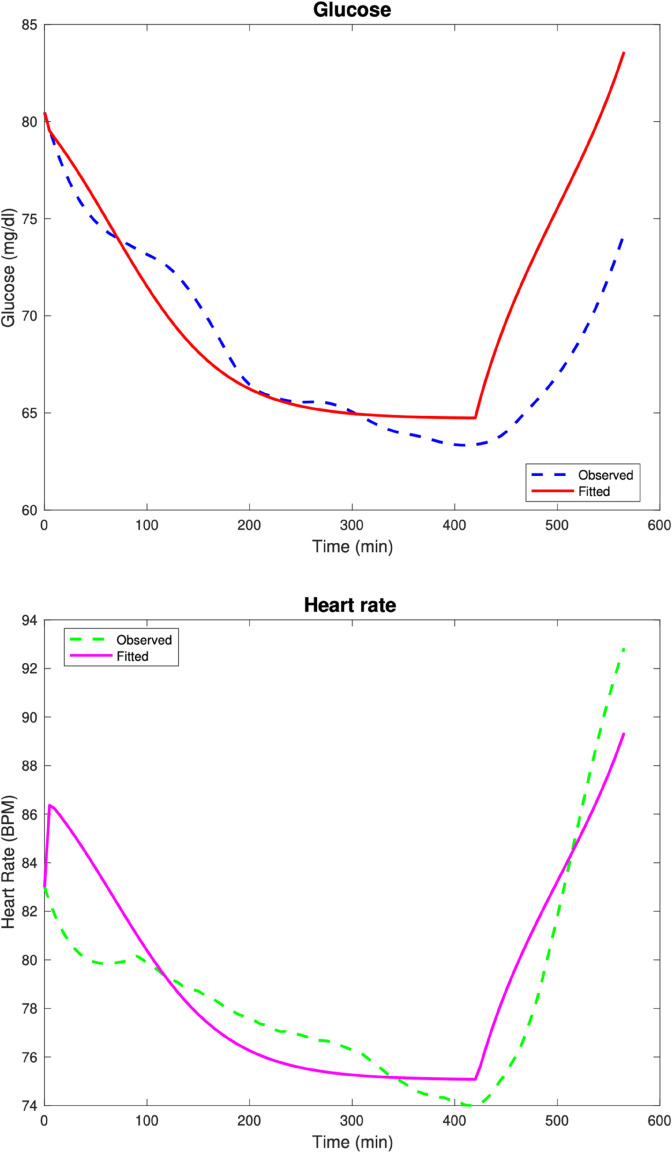
Test Case 3. Data and model fits for a non-diabetic patient with normal risk for T2DM.

Moving onto the real data, in the next test case, we have a patient classified clinically as non-diabetic (i.e., fasting glucose and HbA1c within normal range). The model fits are given in Fig 3. The computed parameters are $R_{g}=0.024,\alpha_{g}=0,k_{g}=0.001,K_{g}=9.998,E_{g}=0.001,\beta_{H}=0.813,H_{v}=30,G_{A}=0.248,G_{W}=300$.

The relative discrete *l*^2^ error percentages for the glucose and heart rate fits are $\|G{\|}_{r}=3.25\%,\;\|H{\|}_{r}=4.01\%$.

**Fig 4:**
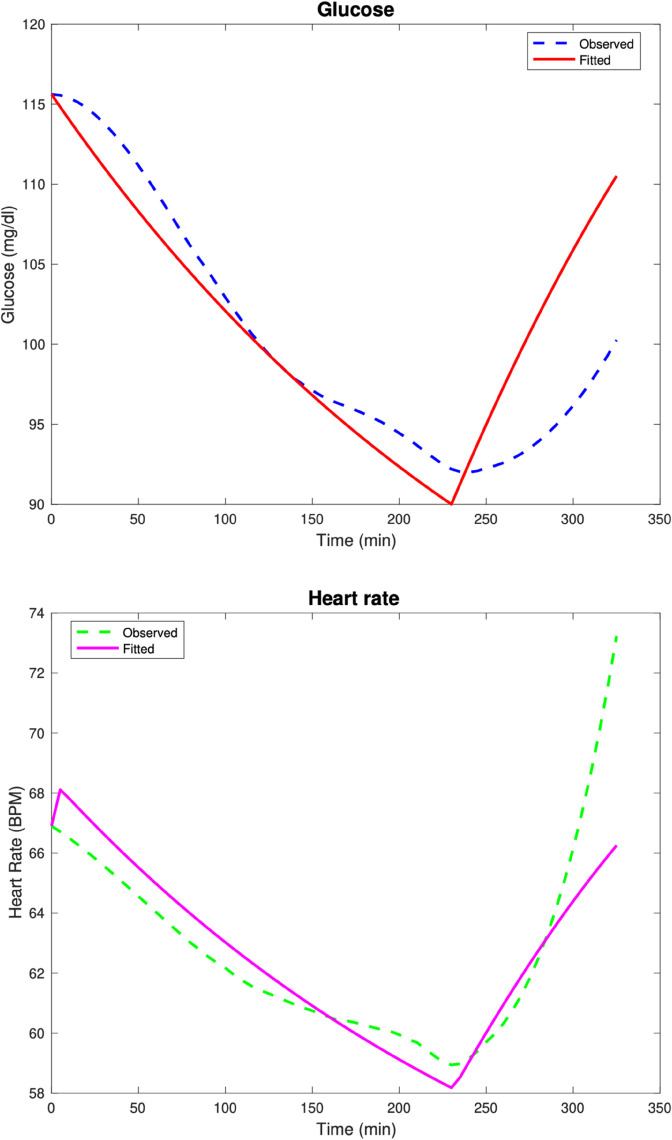
Test Case 4. Data and model fits for a non-diabetic patient at-risk for T2DM.

In the next test case, we have a patient classified clinically as non-diabetic (i.e., fasting glucose and HbA1c within normal range) but is considered at-risk for developing diabetes based on the American Diabetes Association Diabetes Risk Test https://www.niddk.nih.gov/health-information/diabetes/overview/risk-factors-type-2-diabetes/diabetes-risk-test. The model fits are given in Fig 4. The computed parameters are $R_{g}=0.23,\alpha_{g}=0.001,k_{g}=0.001,K_{g}=6.581,E_{g}=0.003,\beta_{H}=0.816,H_{v}=29.988,G_{A}=0.332,G_{W}=298.686$.

The relative discrete *l*^2^ error percentages for the glucose and heart rate fits are $\|G{\|}_{r}=1.45\%,\;\|H{\|}_{r}=4.01\%$.

**Fig 5:**
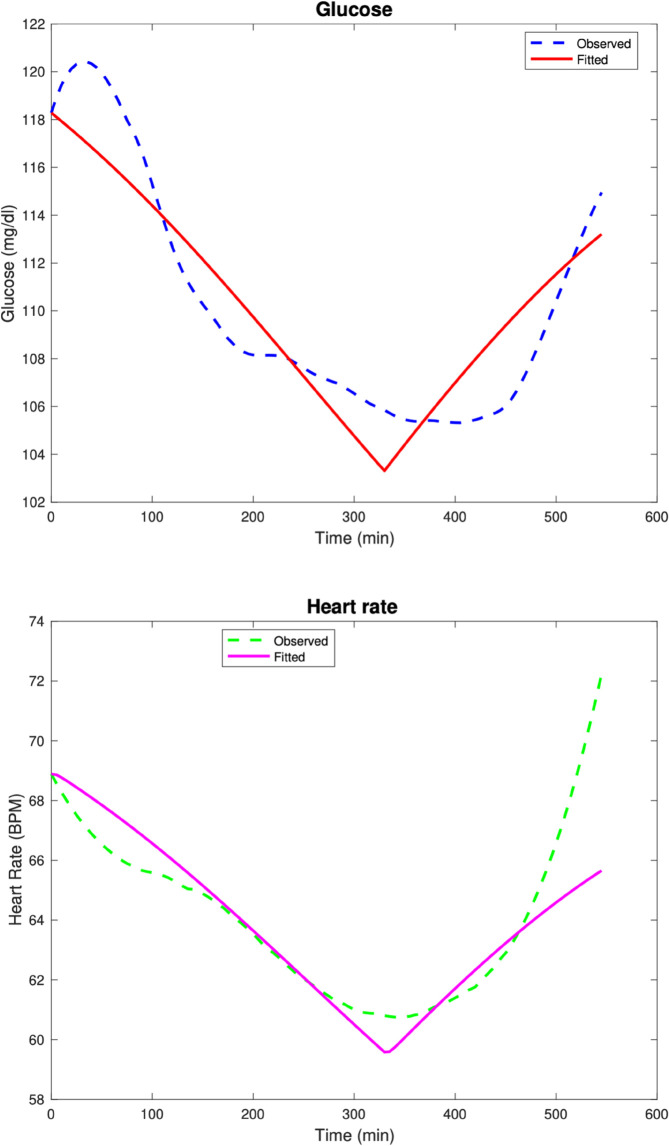
Test Case 5. Data and model fits for a T2DM pre-diabetic patient.

In the fifth test case, we have a patient classified clinically as prediabetic (i.e., fasting glucose and HbA1c within the prediabetic range). The model fits are given in Fig 5. The computed parameters are $R_{g}=9.204,\alpha_{g}=0.007,k_{g}=10,K_{g}=9.790,E_{g}=0.001,\beta_{H}=0.313,H_{v}=20.771,G_{A}=0.106,G_{W}=300$.

The relative discrete *l*^2^ error percentages for the glucose and heart rate fits are $\|G{\|}_{r}=1.8\%,\;\|H{\|}_{r}=2.33\%$.

**Fig 6:**
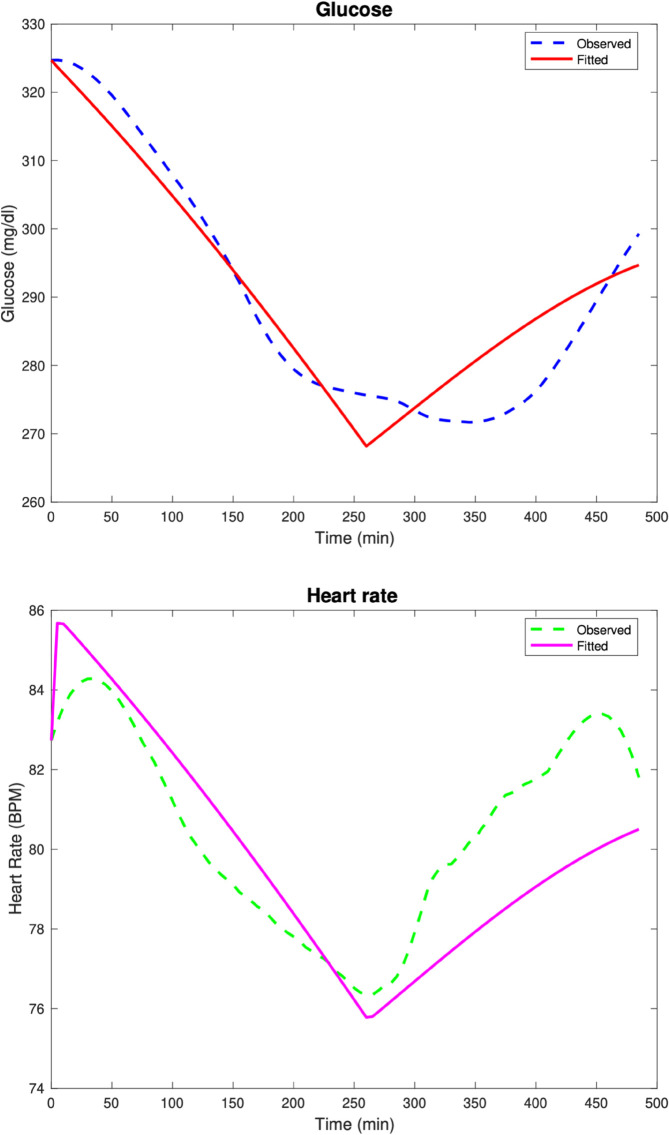
Test Case 6. Data and model fits for a T2DM diabetic patient.

In the last test case, we have a patient classified clinically as diabetic. The model fits are given in Fig 6. The computed parameters are $R_{g}=10,\alpha_{g}=0.003,k_{g}=10,K_{g}=1.866,E_{g}=0.001,\beta_{H}=0.609,H_{v}=30,G_{A}=0.386,G_{W}=300$.

The relative discrete *l*^2^ error percentages for the glucose and heart rate fits are $\|G{\|}_{r}=1.48\%,\;\|H{\|}_{r}=2.82\%$.

Based on the model fits and parameter values, we note that elevated values of *R*_*G*_ (basal glucose production) and $\alpha_{g}$ (amplification of glucose production in response to hormonal cues) indicate that the liver is overproducing glucose under the influence of elevated cardiac activity, during sleep, when it should be suppressed. Simultaneously, a high *k*_*g*_ may signal excessive or dysregulated glucose clearance, possibly reflecting a compensatory or failing effort to manage hyperglycemia. The term $1-\beta_{H}$, representing reduced hormonal damping, points to an impaired ability of the endocrine system to suppress fluctuations in glucose production during rest, a key feature of healthy metabolic control. We also performed a sensitivity analysis using the Latin-hypercube sampling-partial rank correlation method (LHS-PRCC) about parameter means obtained from each of the four test cases [40]. We observed that the parameters $R_{g},\alpha_{g},k_{g},\beta_{H}$ were the only ones that were the most sensitive (with *p*–values less than 0.05) with respect to the maximum glucose level at night and glucose level at the final measurement time. This implies that these parameters form a composite measure of systemic metabolic stress, indicating how much strain the body is under in maintaining glucose balance at night. Thus, we postulate the following risk score:

$$S=10R_{g}+100k_{g}+10000\alpha_{g}+100(1-\beta_{H}).$$ (9)

The coefficients in the risk score definition in (9) were chosen to reflect the relative sensitivities of the model parameters to nocturnal glucose dynamics obtained from the LHS-PRCC analysis and the ranges of the parameters. We first note that the maximum range values of $R_{g},k_{g}$ were 10 times the maximum range value of $\beta_{H}$ and 100 times the maximum range value of $\alpha_{g}$. Thus, weights of $\alpha_{g},\beta_{H}$ in the risk score are atleast assigned 100 and 10 times, respectively, the weights of $R_{g},k_{g}$. Furthermore, *k*_*g*_ and $\alpha_{g}$ exhibited the highest absolute PRCC values with respect to both the peak and terminal glucose levels, indicating that small perturbations in these parameters lead to large deviations in glucose regulation. Consequently, these parameters were assigned an additional multiplicative weight factor of 10 to capture their dominant influence on metabolic dysregulation. The basal glucose production rate *R*_*g*_ showed a moderate sensitivity and was assigned the weight 10, while the heart rate decay parameter $\beta_{H}$, representing parasympathetic recovery, had the weakest direct effect but similar to *R*_*g*_. Thus, the overall weights for $R_{g},k_{g},\alpha_{g},\beta_{H}$ turns out to be 10,100,10000,100, respectively. This scaling ensures that each term contributes proportionally to its physiological significance, thereby maintaining the composite score *S* as both numerically balanced and biologically interpretable across subjects. Based on this score, we formulate the risk categories as follows in Table 3.

**Table 3:** Risk score categories. The categories are based on physiological parameters with cut-off ranges of the risk score and associated interpretations.

Risk score range	Category	Interpretation
*S*>1000	High-risk	Strong metabolic dysregulation
500<S≤1000	Moderate-risk	Early-stage metabolic dysregulation
S≤500	Low-risk	Normal metabolic regulation

The computed risk scores for Test Case 1 synthetic patient is 1153.8 and for Test Case 2 synthetic patient is 123.8. We note from the figures that in case of Patient 1, even though the glucose levels before going to sleep start around 120 mg/dl, the dawn phenomenon spikes the glucose to diabetic levels, which is why the risk score indicates high risk of diabetes. On the other hand, Patient 2 started off with a higher glucose level but then it stabilized even under the dawn phenomenon. The risk score indicated moderate risk but the values were close to the lower range, which means low-to-moderate risk. Some risk factor can be attributed to a slightly elevated heart rate during sleep and while waking up. Both these test cases demonstrates the ability of our model to capture widely varying scenarios.

For the real data cases, the computed risk score for Test Case 3 patient is 29.0, for Test Case 4 patient is 30.8, for Test Case 5 patient is 1230.7 and for Test Case 6 patient is 1169.1. Based on the risk stratification given in Table 3, our model predicted risk-categories are clinically conforming.

## Discussions and future directions

From the results, we observed that even with our simplified model, the fits were very good with relative error percentages within 5%. We also noted that the parameter value of *E*_*g*_ did not vary much from 0.001, even when the range of the parameter was expanded. Thus, we actually set the parameter *E*_*g*_ = 0.001, and so our proposed parameter estimation problem, with loss function as given in (8), only provided outputs of 8 parameters excluding *E*_*g*_.

In the calculation of the risk score, the clinical relevance of the estimated parameters provides important insights into the underlying metabolic and autonomic mechanisms differentiating each risk group. In the non-diabetic patient, the low basal glucose production rate (*R*_*g*_) and near-zero modulation term ($\alpha_{g}$) indicate stable hepatic output and minimal cardiac influence on glucose metabolism, reflecting effective autonomic and hormonal control. For the at-risk individual, a moderate rise in both *R*_*g*_ and $\alpha_{g}$ suggests early sympathetic overactivity and subclinical hepatic overproduction of glucose, an autonomic signature often preceding measurable insulin resistance. In prediabetic and diabetic patients, the markedly elevated *R*_*g*_ and *k*_*g*_ values point to excessive hepatic gluconeogenesis and a dysregulated compensatory increase in glucose clearance attempts, consistent with known features of impaired fasting glucose and β-cell dysfunction. The reduced decay rate parameter ($\beta_{H}$) observed in these cases corresponds to diminished parasympathetic tone and slower return to baseline heart rate, further supporting autonomic imbalance as a co-factor in metabolic dysregulation. The parameters *R*_*g*_ and $\alpha_{g}$ effectively capture hepatic and autonomic drivers of hyperglycemia, while *k*_*g*_ and $\beta_{H}$ reflect compensatory metabolic and cardiovascular mechanisms. This parameter-based interpretation aligns with clinical markers of T2DM progression, such as increased fasting glucose, reduced heart rate variability, and elevated resting heart rate, and supports the proposed risk score as a simplified yet mechanistically grounded index of nocturnal metabolic health.

Thus, our proposed model demonstrates that a minimal two-variable dynamical framework, linking nocturnal glucose and heart rate, can provide a quantitatively interpretable risk measure of metabolic dysregulation. Categorizing T2DM risk among cancer survivors is essential, as it is a common comorbidity linked to increased cancer mortality [2,3]. Prediabetes represents a critical transitional stage that allows for prevention through lifestyle modification [4]. Moreover, incorporating wearable sensors to identify T2DM risk offers a feasible approach that may allow early detection and also address barriers to care access. Our model provides a foundation for personalized intervention, as the risk score can be sensitive to individual changes over time. The derived risk score offers several advantages over conventional indices such as the HOMA-IR or HbA1c-derived metrics. First, it relies on parameters inferred directly from physiological dynamics rather than static or invasive biochemical tests. Second, it can be computed non-invasively from wearable sensor data and thus supports longitudinal, at-home monitoring. Finally, the structure of the risk score allows for clear physiological interpretation, each term corresponds to a measurable biological process (basal glucose production, clearance efficiency, hormonal modulation, and parasympathetic control), making it easily explainable to both clinicians and patients.

The simplicity of this formulation provides an advantage over machine learning–based prediction models that often require high-dimensional data and lack interpretability. Despite its reduced dimensionality, the score successfully stratified subjects across the diabetes continuum, from normal to high-risk, using only nocturnal physiological inputs. This underscores the model’s potential for early-stage, low-cost screening and continuous digital biomarker integration.

A potential future direction is the implication that we will be able to evaluate if any lifestyle changes could lead to an immediate change in the risk score. To achieve this, a further refinement of the proposed mathematical model could be considered. For example, while we focused on glucose and heart rate dynamics during nighttime in the present study, how daytime behaviors (e.g., physical activity and sedentary behavior) might impact nighttime biological metrics can be considered in future model developments.

For our modeling framework, we used a preprocessing technique to take a mean of the data across multiple nights and further smoothen the data to average out the small and rapid fluctuations in the measurements over time. Though our proposed model did capture the average trends well, a more accurate inference can be made by taking into account multiple datasets across each day with rapid fluctuations for each measurement time unit. This leads to accounting for stochastic effects or for simulating multiple trial setups, for which stochastic models are more adept, in the framework of Fokker-Planck or Liouville equations [41–43].

Furthermore, our consideration of a dataset of 4 patients, with distinct diabetic profiles was to demonstrate the feasibility of the proposed mathematical model. Our next step, in a future work, is to rigorously validate this model with bigger patient size and longer monitoring data. Moreover, our current model can also be further extended and personalized by integrating more person-level information (e.g., demographics such as age and weight). One could also introduce lifestyle intervention strategies in the proposed model through the formulation of optimal control frameworks [44]. Since risk for T2DM might be elevated after cancer diagnosis, but is attenuated in later years, another consideration to make the model more tailored for specific type of cancers is to incorporate the time elapsed since cancer survival. In summary, as we are able to track an individual’s behavioral and biological status at high frequency (e.g., at minute-level) over an extended period of time (e.g., over months and even years), we will be able to gain additional insights regarding disease risks and progression by modeling changes in behaviors and biometrics, through the means of such dynamical mathematical modeling frameworks.

## Conclusion

In this work, we have presented a novel dynamical model to capture the behavior of the nocturnal glucose and heart rate and propose a personalized risk-score to classify potential categories of metabolic dysregulation. Our model comprises of a system of ODEs, containing certain parameters that are analyzed for sensitivity to help formulate a risk-score formula. Numerical results with various patient study cases, demonstrate the versatility and robustness of our proposed model. A novelty of our model is the potential scalability and adaptability to future mathematical models that can account for multiple heterogeneitic features and provide personalized lifestyle control mechanisms for improving metabolic regulation.
